# Enhanced detection rate of *Mycoplasma genitalium* in urine overtime by transcription-mediated amplification in comparison to real-time PCR

**DOI:** 10.1186/s12879-023-08499-z

**Published:** 2023-09-04

**Authors:** Nikki Adriaens, Anne-Marije Pennekamp, Alje P. van Dam, Sylvia M. Bruisten

**Affiliations:** 1 Department of Infectious Diseases, Public Health Service of Amsterdam, Amsterdam, The Netherlands; 2 Department of Medical Microbiology, Amsterdam University Medical Center, Amsterdam, The Netherlands

**Keywords:** Sexually transmitted infection, *Mycoplasma genitalium*, Molecular diagnostic techniques, Nucleic acid amplification test, Transcription-mediated amplification, Antimicrobial resistance

## Abstract

**Background:**

Diagnosis of infected individuals with *Mycoplasma genitalium* (MG) is often performed by real-time PCR or transcription-mediated amplification (TMA). A limitation of the MG-TMA assay is the relatively short time span of 24 h in which the collected urine is required to be transferred into a Urine Specimen Transport Tube, according to the manufacturer’s guidelines. If not transferred within 24 h, the manufacturer’s claimed sensitivity cannot be guaranteed anymore, and samples may instead be tested with an in-house validated real-time PCR, despite its recognized lower sensitivity. This study aimed to validate an exception to the sample transport and storage conditions of the MG-TMA assay as set by the manufacturer, being the prolongation of the acceptable testing time limit of 24 h.

**Methods:**

From June to December 2022, first-void urines were collected from clients attending the clinic for sexual health in Amsterdam, the Netherlands. Urine samples that tested positive for MG by TMA assay at the day of collection were concomitantly stored at room (18–24 °C) and refrigerator temperature (4–8 °C) for 15 days. The stored urine samples were tested with both an in-house validated real-time PCR and MG-TMA assay after transfer of the original urine samples to the respective test tubes at 3, 7, 12 and 15 days post collection.

**Results:**

In total, 47 MG-positive urine samples were collected, stored and tested for MG by real-time PCR and TMA assays. After storage at room temperature, the MG-detection rate by TMA was significantly higher compared to real-time PCR, at days 0 (*p* ≤ 0.001), 7 (*p* ≤ 0.001) and 12 (*p* < 0.05). After storage at refrigerator temperature, the MG-detection rate determined by TMA assay was significantly enhanced in comparison with real-time PCR at days 3 (*p* < 0.01), 7 (*p* ≤ 0.001) and 15 (*p* < 0.01).

**Conclusions:**

This validation study showed that the MG**-**TMA assay has a superior detection rate in urine compared to real-time PCR, up to 15 days post sample collection and irrespective of storage temperature. Accepting urines older than 24 h to be tested by TMA will improve clinical diagnosis of MG infections.

**Supplementary Information:**

The online version contains supplementary material available at 10.1186/s12879-023-08499-z.

## Background

*Mycoplasma genitalium* (MG) is a common causative pathogen for urethritis in males, and is potentially associated with adverse reproductive consequences in females, including pelvic inflammatory disease [1]. According to the 2021 European guideline on the management of MG infections [2], azithromycin and moxifloxacin are considered as the first- and second-line treatment regimes, respectively, in individuals with an uncomplicated MG infection. Due to the emerging antimicrobial resistance in MG worldwide, adequate diagnosis of individuals infected with MG is important for optimal antimicrobial stewardship.

The most frequently performed nucleic acid amplification tests for the identification of MG in urine samples include real-time PCR and Aptima transcription-mediated amplification (TMA) assays [3, 4]. The MG-TMA assay works by targeting rRNA, which is present in numerous copies per cell, thereby contributing to its increased detection rate compared to the MG real-time PCR assay, targeting the single-copy DNA target gene *MgPa* coding for the MG adhesin protein [4, 5].

A limitation of the commercially available MG-TMA assay is the relatively short time span of 24 h in which the collected urine is required to be transferred into an Aptima Urine Specimen Transport Tube, according to the manufacturer’s instructions. For most non-clinic based laboratories, the transportation time between collection and testing of urine samples may be longer than 24 h, particularly in case of self-tests or tests collected at a general practitioners office. The manufacturer’s instructions indicate to deny testing by TMA assay in that case, since the claimed sensitivity cannot be guaranteed anymore. Consequently, an in-house validated real-time PCR without a restriction for transportation time may be used instead to detect MG, despite the generally recognized lower sensitivity of real-time PCR relative to TMA [6–9]. In this study, it was aimed to validate an exception to the sample transport and storage conditions of the MG-TMA assay as set by the manufacturer, being the prolongation of the acceptable testing time limit of 24 h.

## Methods

### Sample collection and handling

First-void urines were collected from clients attending the center for sexual health (CSH) in Amsterdam, the Netherlands, from June to December 2022. Urine samples that tested positive at day of collection (day 0) for MG by TMA assay (Aptima *Mycoplasma genitalium* assay; Hologic Inc. San Diego, CA, US) were anonymized and included in the study. A time scheme with days of testing was designed (Table S1) to ensure a minimum of twenty urine samples tested per timepoint. The included samples were split into two equal volumes of at least 10 mL each and stored at both room (18–24 °C) and refrigerator temperature (4–8 °C) for a total duration of 15 days. Storage at room temperature served as a simulation of transportation and storage at refrigerator temperature as a control for accelerated degradation. Subsequently, stored urine samples were tested by both the in-house validated real-time PCR [4, 10] and the MG-TMA assay [11]. Prior to each test at days 3, 7, 12 and 15 post collection, urine samples were transferred from the original urine container to the respective test tubes.

### qPCR assay

Per test, MG DNA was extracted from 1 mL of urine sample using isopropanol precipitation and subsequently tested by the in-house TaqMan *MgPa* real-time PCR in order to detect MG, as previously described [4, 10].

### TMA assay

For detection of MG by TMA, 2 mL of urine sample was transferred to an Aptima Urine Specimen Transport Tube (Hologic Inc. San Diego, USA), which additionally contained 2 mL of specimen transport medium. From each Aptima Urine collection kit, 0.4 ml of the mixture was dispensed to the multi-tube unit to extract, amplify and detect MG 16 S rRNA, as previously described [11]. The TMA assay was performed on a Panther system according to the manufacturer’s instructions.

### Ethics statement

Clients of the CSH outpatient clinic were informed of the “opt-out” system regarding research on remnants of client material. Material from clients was only included in this study if clients did not opt-out. All data were fully anonymized before assessment. Results of MG testing were not disclosed to healthcare professionals or clients. The requirement for ethical approval and informed consent was waived by the Institutional review board which is the Medical Ethics Committee of the Academic Medical Center in Amsterdam because of the retrospective nature of the study. All methods were performed in accordance with the relevant guidelines and regulations.

### Statistical analysis

Cohen’s Kappa statistic (κ) was used to assess the concordance between MG-detection rate determined by real-time PCR and TMA assays per individual timepoint. McNemar’s test was used to evaluate significant differences (*p*-value < 0.05) in the detection rates between groups. Statistical calculations and analyses were performed in SPSS Statistics (version 26.0, IBM, Armonk, NY, USA). GraphPad Prism (version 8, GraphPad Software, La Jolla California, USA) was used to create graphs.

## Results

### Detection rate of MG by real-time PCR and TMA assays

**Fig. 1:**
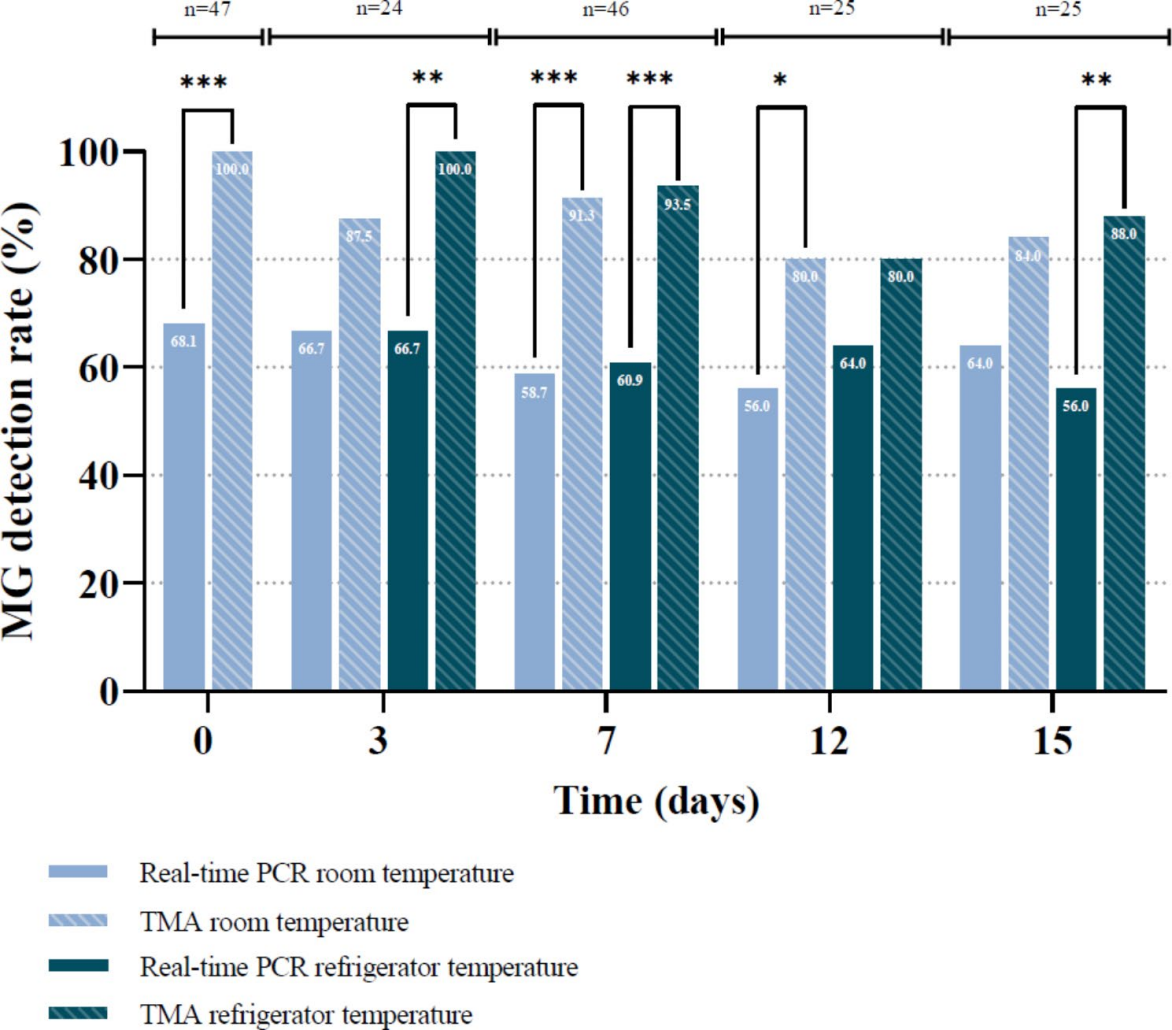
Detection rate (%) of *Mycoplasma genitalium* (MG) in urine samples overtime. MG-positive urines were determined by real-time PCR and TMA assays after storage at room and refrigerator temperature for a total duration of 15 days. Data shown are percentages of MG-positive urines per total number of urines included, as specified in the respective bars. McNemar’s test was performed to compare MG-detection rate (%) between groups (*, *p* < 0.05; **, *p* < 0.01; ***, *p* ≤ 0.001)

In total, 47 MG-positive urine samples with a minimal volume of 30 mL were collected and included. Among the TMA-positives at the day of collection (day 0), 32/47 (68.1%) tested positive for MG by real-time PCR (Fig. 1). After storage at room temperature, 21/24 (87.5%), 42/46 (91.3%), 20/25 (80.0%) and 21/25 (84.0%) urine samples tested positive for MG by TMA assay, at days 3, 7, 12 and 15. For the real-time PCR assay, MG was detected in 16/24 (66.7%), 27/46 (58.7%), 14/25 (56.0%) and 16/25 (64.0%) urine samples at days 3, 7, 12 and 15, respectively. The detection rate of MG by TMA assay was significantly higher compared to real-time PCR, at days 0 (*p* ≤ 0.001), 7 (*p* ≤ 0.001) and 12 (*p* < 0.05). After storage in the refrigerator, detection of MG by TMA was observed in 24/24 (100%), 43/46 (93.5%), 20/25 (80.0%) and 22/25 (88.0%) for days 3, 7, 12 and 15, respectively (Fig. 1). The real-time PCR assay detected MG in 16/24 (66.7%), 28/46 (60.9%), 16/25 (64.0%) and 14/25 (56.0) at days 3, 7, 12 and 15, respectively. The MG-detection rate determined by TMA assay was significantly enhanced in comparison to real-time PCR, at days 3 (*p* < 0.01), 7 (*p* ≤ 0.001) and 15 (*p* < 0.01). There were no significant differences in detection rates of MG observed between urines stored at room or refrigerator temperature, for neither real-time PCR nor TMA assays.

### Concordance of real-time PCR and TMA assay results after storage of urine samples at room temperature overtime

The highest concordance between real-time PCR and TMA assay results was for urine samples tested at day 15 (κ = 0.506; *p* < 0.01 (Table S2A)). There was no significant concordance observed between real-time PCR and TMA assay results for the urines tested at day 7 (κ = 0.035; *p* > 0.05).

### Concordance of TMA and real-time PCR assay results after storage of urine samples at refrigerator temperature overtime

The highest concordance between real-time PCR and TMA assay results was for urine samples tested at day 12 (κ = 0.615; *p* ≤ 0.001 (Table S2B)). There was no significant concordance observed between real-time PCR and TMA assay results for the urines tested at day 7 (κ = 0.089; *p* > 0.05).

## Discussion

In the current study, we explored the impact of the duration of transport and storage conditions upon real-time PCR and MG-TMA assay performance. This aimed to validate an exception to the sample transport and storage conditions of the MG-TMA assay as set by the manufacturer, being the prolongation of the acceptable testing time limit of 24 h.

To our knowledge, this was the first study to compare detection rates of MG by real-time PCR and TMA in urine samples over a prolonged period post collection. In 2015, an overall MG prevalence of 3.1% was determined using MG real-time PCR in clients visiting the CSH in Amsterdam, the Netherlands [12]. Later on in 2021, the overall prevalence in a comparable clinical population was increased to 13.8%, this time by testing using the MG-TMA assay [13]. This increase in MG prevalence in a similar clinical population could be partially explained by the overall increase in the number of individuals infected, however the observation that TMA is generally a more sensitive diagnostic tool for the detection of MG than real-time PCR may also have contributed. Several studies evaluated the difference in sensitivity of *MgPa* real-time PCR and MG-TMA immediately after collection of clinical samples. In 2016, Tabrizi et al. [3] found a sensitivity of 91.3% and 100% respectively for real-time PCR and TMA, after testing a total of 1080 urine samples for MG positivity. Also a study conducted by Unemo et al. [14] showed that the TMA assay was able to detect MG in clinical specimens with an improved sensitivity compared to real-time PCR, including urine, rectal-, urethral, vaginal, cervical and pharyngeal swabs. The aforementioned comparisons between real-time PCR and TMA assays did however not look at a potential decrease in detection rate overtime, which is particularly important to know in the diagnostic setting. In laboratories that are non-clinic based, the transportation time may be extended up to several days. According to the manufacturer’s instructions of the Aptima MG-TMA assay, urine samples received more than 24 h after collection should not be tested. Our comparison of the MG real-time PCR and MG-TMA assay showed that up to 15 days post sample collection, the detection rate of MG by TMA is still enhanced compared to real-time PCR as it was at the first day of collection.

The difference in sensitivities between the MG real-time PCR and MG-TMA assays may be explained by their distinctive amplification mechanisms. PCR includes the doubling of target DNA in each of the 45 amplification cycles, while during TMA hundreds to thousands of target RNA copies are generated isothermally, which all can consequently serve as transcriptional templates. This superior TMA sensitivity was described for MG [8] but also for other sexually transmitted pathogens [6, 7] and viruses [9].

The short time period of maximum 6 h in which urine samples were received post collection is a strength of this study, since this established a reliable simulation of long transportation times. A limitation is the fluctuating number of tests performed per timepoint due to the practical restriction of no routine testing on weekend days or official holidays. However, sample sizes were still large enough to confirm our finding demonstrating that urine samples can be tested with a relatively high detection rate up to 7 days of 80–100% by MG-TMA assay in urines received later than 24 h post collection. A disclaimer will however still be added in the routine setting for all MG-negatively tested urine samples received later than 24 h post collection to warn for the overall decreasing detection rate overtime.

## Conclusion

This validation study showed that the MG-TMA assay has a superior detection in urine compared to the MG real-time PCR, up to 15 days post sample collection and irrespective of storage temperature. Urine samples received later than 24 h post collection can be tested up to 7 days with a relatively high detection rate using the MG-TMA assay. Accepting urines older than 24 h to be tested by TMA will improve clinical diagnosis of MG infections. Therefore, it is recommended for diagnostic laboratories to still test urines received later than 24 h post collection, noting to users the impact that this may have upon assay sensitivity.

### Electronic supplementary material

Below is the link to the electronic supplementary material.

Supplementary Material 1

Supplementary Material 2

Supplementary Material 3

## Data Availability

All data generated or analysed during this study are included in this published article and its supplementary information files.

## Abbreviations

CSH: center for sexual health
MG: Mycoplasma genitalium
TMA: transcription-mediated amplification
