# Supplementary material for: Enhanced detection rate of Mycoplasma genitalium in urine overtime by transcription-mediated amplification in comparison to real-time PCR
Source: BMC Infect Dis. 2023 Sep 4;23:574. doi: 10.1186/s12879-023-08499-z (PMC10476297; doi:10.1186/s12879-023-08499-z)
Supplement: Supplementary file 1 — Supplementary Material 1 [file 12879_2023_8499_MOESM1_ESM.docx]

**Table S1.** Time scheme specifying days of testing.

| Day of collection (day 0) | Day 3 | Day 7 | Day 12 | Day 15 |
| --- | --- | --- | --- | --- |
| Monday | Thursday | Monday | Saturday^a^ | Tuesday |
| Tuesday | Friday | Tuesday | Sunday^a^ | Wednesday |
| Wednesday | Saturday^a^ | Wednesday | Monday | Tuesday |
| Tuesday | Sunday^a^ | Tuesday | Tuesday | Friday |
| Friday | Monday | Friday | Wednesday | Saturday^a^ |

^a^ Samples were not routinely tested on weekend days or official holidays.
