# Supplementary material for: Enhanced detection rate of Mycoplasma genitalium in urine overtime by transcription-mediated amplification in comparison to real-time PCR
Source: BMC Infect Dis. 2023 Sep 4;23:574. doi: 10.1186/s12879-023-08499-z (PMC10476297; doi:10.1186/s12879-023-08499-z)
Supplement: Supplementary file 2 — Supplementary Material 2 [file 12879_2023_8499_MOESM2_ESM.docx]

**Table S2A.** Concordance of qPCR and TMA assay results among urine samples stored at room temperature overtime.

| **TMA assay result** | **PCR assay result** | | | **Concordance (%)** | **κ-value** | ***p*-value** |
| --- | --- | --- | --- | --- | --- | --- |
|  | **Positive** | **Negative** | **Total** |  |  |  |
| **Day 3**  **Positive**  **Negative**  **Total** | 16  0  16 | 5  3  8 | 21  3  24 | 79.2 | 0.444 | 0.009 |
| **Day 7**  **Positive**  **Negative**  **Total** | 25  2  27 | 17  2  19 | 42  4  46 | 58.7 | 0.035 | 0.712 |
| **Day 12**  **Positive**  **Negative**  **Total** | 14  0  14 | 6  5  11 | 20  5  25 | 76.0 | 0.483 | 0.005 |
| **Day 15**  **Positive**  **Negative**  **Total** | 16  0  16 | 5  4  9 | 21  4  25 | 80.0 | 0.506 | 0.004 |
