# Supplementary material for: Enhanced detection rate of Mycoplasma genitalium in urine overtime by transcription-mediated amplification in comparison to real-time PCR
Source: BMC Infect Dis. 2023 Sep 4;23:574. doi: 10.1186/s12879-023-08499-z (PMC10476297; doi:10.1186/s12879-023-08499-z)
Supplement: Supplementary file 3 — Supplementary Material 3 [file 12879_2023_8499_MOESM3_ESM.docx]

**Table S2B.** Concordance of qPCR and TMA assay results among urine samples stored at refrigerator temperature overtime.

| **TMA assay result** | **PCR assay result** | | | **Concordance (%)** | **κ-value** | ***p*-value** |
| --- | --- | --- | --- | --- | --- | --- |
|  | **Positive** | **Negative** | **Total** |  |  |  |
| **Day 3**  **Positive**  **Negative**  **Total** | 16  0  16 | 8  0  8 | 24  0  24 | 66.7 | NA^a^ | NA^a^ |
| **Day 7**  **Positive**  **Negative**  **Total** | 27  1  28 | 16  2  18 | 43  3  46 | 63.0 | 0.089 | 0.312 |
| **Day 12**  **Positive**  **Negative**  **Total** | 16  0  16 | 4  5  9 | 20  5  25 | 84.0 | 0.615 | 0.001 |
| **Day 15**  **Positive**  **Negative**  **Total** | 14  0  14 | 8  3  11 | 22  3  25 | 68.0 | 0.296 | 0.037 |

^a^ κ-value and *p*-value could not be determined since TMA assay result is a constant variable.
